# Multiple cardiac papillary fibroelastomas: a case report and review of the literature

**DOI:** 10.3389/fcvm.2025.1455947

**Published:** 2025-05-30

**Authors:** Mostafa Ali, Mohammad Alomari, Magdy M. El-Sayed Ahmed, Pankaj Garg, Anthony N. Pham, Si M. Pham

**Affiliations:** ^1^ Department of Cardiothoracic Surgery, Mayo Clinic, Jacksonville, FL, United States; ^2^ Department of Surgery, Zagazig University Faculty of Human Medicine, Zagazig, Egypt

**Keywords:** cardiac papillary fibroelastomas, cardiac tumor, valvular abnormalities, cardiac surgery, management of heart tumors

## Abstract

Cardiac papillary fibroelastomas (PFEs) are the most common benign cardiac tumors and are typically solitary. PFEs affecting both sides of the heart are exceptionally rare, with only four cases reported in the literature. Herein, we report a case of a 63-year-old male presenting with signs and symptoms of embolic strokes and an embolism in the coronary arteries. An echocardiogram showed multiple masses on both the mitral and tricuspid valve leaflets. Because of the risk of embolism, he underwent successful valve-sparing surgical resection without complications. The follow-up echocardiogram at 6 months showed no recurrence and competence of both the mitral and tricuspid valves with minimal regurgitation.

## Background

Primary cardiac tumors (PCTs) are rare; most of the epidemiological data on these tumors are derived from autopsy studies, which estimate an incidence of 0.002%–0.3% and a prevalence of 0.001%–0.03% (1). Most cases are secondary to metastasis, while PCTs account for less than 5%. Among PCTs, benign types are predominant. Within this category, myxomas are most commonly identified in autopsy series, whereas papillary fibroelastomas (PFEs) are more frequently reported through echocardiography and pathology (2). Although the exact prevalence of PFEs remains uncertain, recent investigations estimate it to be approximately 10%, with tricuspid PFEs accounting for 6%–15% of cases (3).

PFEs are small, benign endocardial lesions, primarily valvular, and are clinically significant due to their potential to cause embolic events (4). Cardiac PFEs are typically solitary, with multiple PFEs constituting less than 10% of cases. They usually present on the valvular endocardium of the left side of the heart (5). Multiple PFEs involving both sides of the heart are extremely rare, with only four reported cases in the literature (4, 6–8).

In this report, we present a case of multiple PFEs affecting both the mitral and tricuspid valves in a patient who presented with signs and symptoms of emboli in the coronary arteries and brain.

## Case presentation

**Figure 1:**
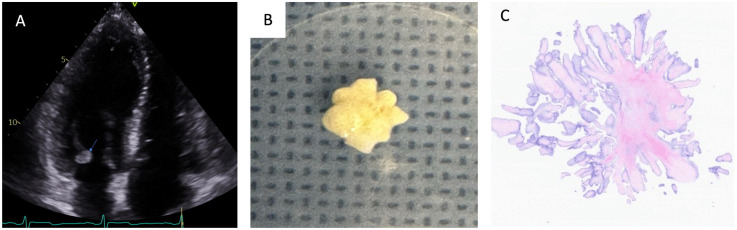
Fibroelastoma on the posterior leaflet of the mitral valve. Transthoracic echocardiography revealed a round, mobile mass (indicated by the arrow) adherent to the posterior leaflet of the mitral valve **(A)**. A macroscopic image shows a gray-white lesion measuring 1 cm in its greatest dimension **(B)**. Light microscopy (hematoxylin and eosin staining at 40× magnification) demonstrated papillary fibroelastomas with many avascular fonds lined by endothelial cells **(C)**.

**Figure 2:**
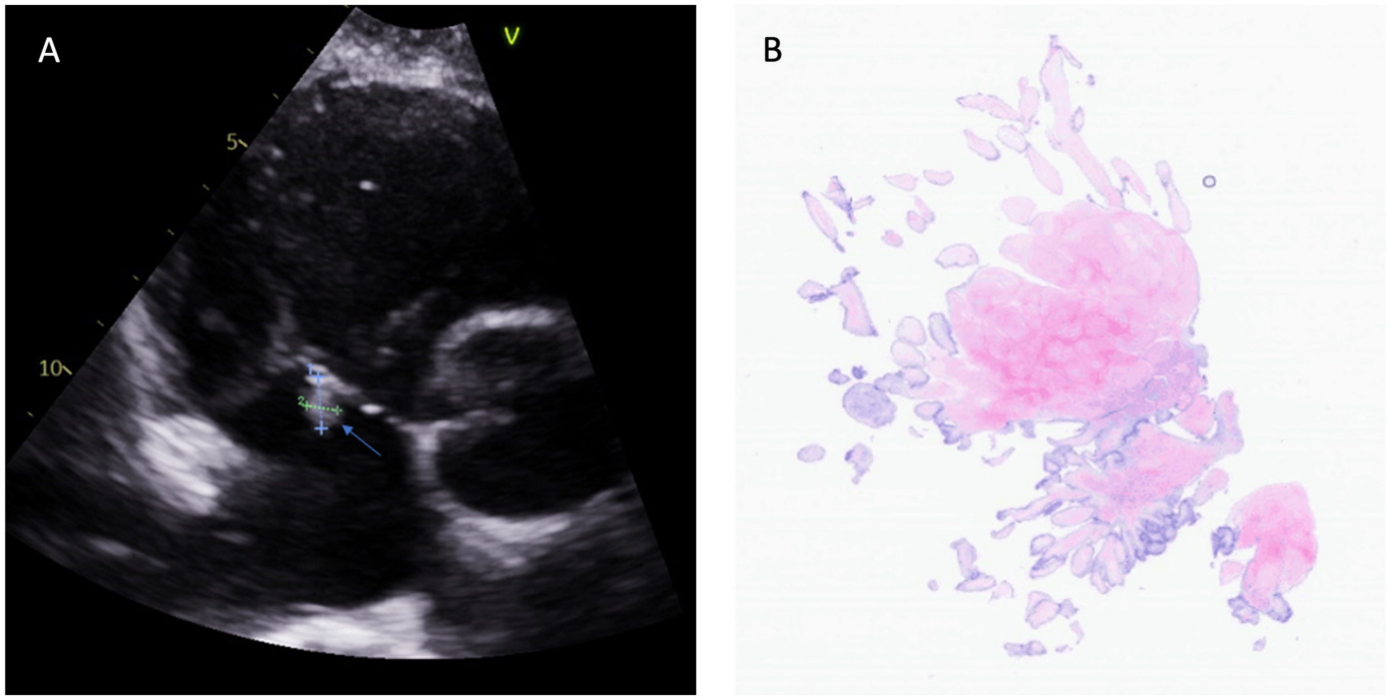
Fibroelastoma on the septal leaflet of the tricuspid valve. **(A)** Transthoracic echocardiography identified a small, mobile mass on the septal leaflet of the tricuspid valve (indicated by the arrow). **(B)** Light microscopy (hematoxylin and eosin staining of the mass at 40× magnification) revealed the characteristics of the papillary fibroelastomas.

A 63-year-old male with a history of hyperlipidemia and tobacco use presented with chest pain for 3 days. The patient was afebrile, and the clinical examination was unremarkable. An electrocardiogram (EKG) revealed ST elevation in leads V3 and V5, and an echocardiogram showed a mobile mass on the posterior leaflet of the mitral valve and two mobile masses on the tricuspid valve: one on the septal leaflet and one in the anterior leaflet (Figures 1, 2). There was minimal mitral and tricuspid regurgitation. Endocarditis vegetations were suspected and intravenous cefazolin was started (2 g/8 h). Brain computed tomography (CT) was obtained as part of the endocarditis evaluation and showed a few areas of decreased attenuation within the subcortical white matter concerning for acute/subacute infarcts. The brain magnetic resonance imaging (MRI) showed multiple small brain infarcts. The patient was thought to have old embolic strokes and transient embolic thrombi.

Laboratory results were within normal limits: hemoglobin 14.6 g/dl, leukocytes 5.5 × 10^3^/µl, platelets 248 × 10^3^/µl, C-reactive protein (CRP) <3 mg/L, erythrocyte sedimentation rate (ESR) 3 mm/h, aspartate aminotransferase (AST) 26 U/L, creatinine 0.84 mg/dl, lactate 1.6 mmol/L, and troponin 8 ng/L. Blood cultures showed no growth. The coronary angiogram revealed eccentric calcified plaques in the proximal and mid segments of the left anterior descending artery (LAD), resulting in mild luminal stenosis, while the distal LAD remained patent. The left circumflex artery (LCX) exhibited calcified and mixed plaques in the proximal segment with mild stenosis. The right coronary artery (RCA) was unremarkable (Supplementary Videos S1, S2). He was placed on systemic anticoagulation—apixaban 5 mg twice a day—and was referred for surgical removal of the cardiac masses. The patient was operated on through median sternotomy on cardiopulmonary bypass. Intraoperative findings include two masses attached to the tricuspid valve: one 8 mm × 5 mm mass was attached with a stalk to the septal leaflet and a second 5 mm × 5 mm mass was attached to the posterior leaflet near the anteroposterior commissure. Both masses were excised.

In the left side of the heart, there were three discrete masses found on the mitral valve leaflets: a 10 mm × 10 mm on the posterior mitral leaflet at the P2 level, a 5 mm × 7 mm mass on the posterior mitral leaflet at the P3 level, and a third sessile mass (2 mm × 5 mm) on the anterior mitral leaflet at A2 level. In addition, there were multiple micronodules, with a sandpaper appearance on the atrial surface of the anterior leaflet of the mitral valve, covering approximately 20% surface of the anterior leaflet. Moreover, micronodules covered the tip of the medial and lateral papillary muscles. These nodules were too small to resect.

Histopathology confirmed a diagnosis of fibroelastoma (Figures 1, 2). The postoperative course was unremarkable, and the patient was discharged from the hospital on Aspirin 325 mg/day on postoperative day 4. A follow-up echocardiogram at 1 year revealed competent cardiac valves and no residual mass (Supplementary Video S3).

## Discussion

Multiple PFEs involving both sides of the heart are rare; only four reported cases have been reported (Table 1). All these patients were female and were older than 40 years of age at the time of diagnosis. It has been suggested that multiple PFEs may emerge due to stimulus-driven responses to heart surgeries and abnormalities such as hypertrophic obstructive cardiomyopathy (HOCM) (8). Further, a genetic association with multiple PFEs has also been recently reported. Muyldermans et al. reported MYBPC3 gene mutation in a patient with HOCM and multiple PFEs (9).

**Table 1:** Characteristics of the reported cases of multiple cardiac papillary fibroelastomas.

Reference	Age/sex	Presentation	Cardiac history	Embolic events	No. of valves affected	No. of masses	Valvular insufficiency	Other surgical procedures	Follow-up (months/recurrence)
Patel et al. (4)	76 years/F	Fatigue, pedal edema, and exertional dyspnea	Bradycardia	None	Two: RA, TV, AV, and LV	10	None	None	Not reported
Vittala et al. (6)	53 years/F	Asymptomatic	MV prolapse	None	Three: MV, TV, and PV	3	MV and AV regurgitation	MV annuloplasty, AV repair	1/No
Iosifescu et al. (7)	63 years/F	Embolic stroke	None	Embolic stroke	Three: Chords, MV, TV, PV, and LV endocardium	10	MV and AV regurgitation	MV and TV replacement	Not reported
Kumar et al. (8)	41 years/F	Chest pain, exercise intolerance, and palpitation	Noonan syndrome and HOCM	Pulmonary embolism	Zero: LV and RV	35–40	Moderate PV and TV regurgitation	PV replacement	Not reported
Current case	63 years/M	Asymptomatic	None	Systemic embolism (brain and coronary artery)	Two: MV and TV	5, multiple small nodules	None	None	6/No

MV, mitral valve; TV, tricuspid valve; PV, pulmonary valve; AV, aortic valve; LV, left ventricle; HOCM, hypertrophic obstructive cardiomyopathy, RA, right atrium; RV, right ventricle.

Histopathologically, cardiac PFEs are characterized by an avascular collagenous core, which consists of proteoglycans, elastic fibers, fibroblasts, and occasionally spindle cells and calcification, enveloped by a single layer of endocardial cells. Grossly, they present as mobile papillary projections attached to a stalk (10).

They are often found incidentally through imaging, surgery, or post mortem examinations. While most cases are asymptomatic, symptomatic patients may experience embolization-related complications, with stroke being the most common presentation. In some instances, chest pain due to myocardial infarction or acute abdominal pain due to acute mesenteric ischemia may be their first presentation. Other features, including heart murmurs, dyspnea, pulmonary hypertension, and arrhythmia, were also reported (11, 12). In the four reported cases of multiple PFEs that involved both sides of the heart, one patient was asymptomatic and diagnosed incidentally during routine follow-up (6). Two patients experienced embolic events [pulmonary (8) and brain (7)], and three patients had a history of cardiac disorders, mitral prolapse (6), bradyarrhythmia (4), and Noonan syndrome with HOCM (8).

Echocardiography is highly sensitive and specific, making it the preferred diagnostic tool. PFEs appear as a small (usually not exceeding 20 mm) endocardial mass that is homogenous, well-defined, echo-dense, and mobile with uniform or irregular borders and are traditionally associated with single or multiple stalks. The small size is suggestive of a thrombus or vegetation as a differential diagnosis (5). Cardiac CT and MRI can be used as adjunctive diagnostic tests.

Four patients, including ours, exhibited two or three (6, 7) valves affected by PFEs, and the number of masses was <10 (3, 6, 7). There was no valvular involvement in one patient despite 35–40 masses in the left and right ventricles (8). Even though the aortic valve is the most common valve affected with PFEs (12), in patients with multiple bilateral cardiac PFEs, aortic valve involvement has been reported in only one case (4). In patients with numerous PFEs, significant valvular regurgitation may occur and need to be addressed during surgery. In our review, three patients had significant regurgitation in their aortic (6), mitral (6, 7), tricuspid (7, 8), and pulmonary (8) valves, and the affected valve was either repaired or replaced.

Because PFEs carry a potential risk of complications, especially embolic events, complete surgical excision of all the masses is recommended (13). However, it may not be possible to safely remove all the masses without disrupting the heart's integrity, as reported by Patel et al. (4). Furthermore, due to the lack of data, the long-term outcome is unknown. Hence, after surgery, all patients with multiple cardiac PFEs should be closely followed with serial echocardiography to detect any growth of the residual masses or recurrence. Surgical approaches depend on the site, size, number of masses, and surgeon preference. There is no consensus regarding the management of asymptomatic and small right-sided PFEs. Some have suggested regular follow-up, while others have recommended surgical excision (14, 15). For solitary or multiple PFEs involving aortic valves, a minimally invasive technique is gaining popularity; however, median sternotomy remains the preferred approach for multiple PFEs involving both sides of the heart (16). All the patients in the present review underwent surgical resection without major complications; however, follow-up data regarding recurrence were not reported for two of the patients (4, 7). In patients with contraindications to surgery, antiplatelet and/or anticoagulation therapy are recommended with no supporting data (17).

In summary***,*** multiple PFEs that occur on both sides of the heart are rare, but systemic embolization occurs frequently, therefore, surgical removal is recommended to avoid complications. Ongoing, routine follow-up is recommended to monitor for recurrence.

## Data Availability

The original contributions presented in the study are included in the article/Supplementary Material, further inquiries can be directed to the corresponding author.

## Supplementary material

Supplementary Video S1 Coronary angiogram: The Left Anterior Descending (LAD) artery exhibits eccentric calcified plaques in the proximal and mid segments, causing mild luminal stenosis, with the distal LAD remaining patent and free of stenosis. The Left Circumflex (LCX) artery demonstrates calcified and mixed plaques in the proximal segment with mild luminal stenosis.

Supplementary Video S2 Coronary angiogram: The Right Coronary Artery (RCA) appears normal.

Supplementary Video S3 Echocardiogram at the 1-year postoperative follow-up showed normal valvular function with no evidence of residual mass or recurrence.
